# The impact of aminoglycoside exposure on soil and plant root-associated microbiota: a meta-analysis

**DOI:** 10.1186/s13750-025-00365-6

**Published:** 2025-07-10

**Authors:** Jessica L. Coates, Ashanti J. Lawson, Kathleen Bostick, Mentewab Ayalew

**Affiliations:** Biology Department, Spelman College, 350 Spelman Lane, Atlanta, GA 30314 USA

**Keywords:** Microbial composition, Resistome, Pathogen suppression, Plant root microbiota

## Abstract

**Background:**

Exposure to aminoglycosides, a class of potent bactericidal antibiotics naturally produced by soil microorganisms and commonly used in agriculture, has the potential to cause shifts in the population dynamics of microorganisms that impact plant and soil health. In particular, aminoglycoside exposure could result in alterations of the soil and plant root-associated bacterial species diversity and richness due to their potent inhibitory action on microbial growth, the creation of selective conditions for the proliferation of antibiotic-resistant bacteria, or a reduction in the ability to suppress soil pathogens. Previous studies have attempted to understand the relationship between aminoglycoside exposure and the plant-associated microbiota with varying results. Thus, this systematic review aims to survey all relevant published data to answer the question, “What is the impact of aminoglycoside exposure on the soil and plant root-associated microbiota?”

**Methods:**

We searched 5 academic databases and 1 specialist organization database for scientific journal publications written in any language. Articles were included based on the criteria described in Coates et al., 2022. Included studies were subject to critical appraisal using the CEE Critical Appraisal Tool Version 0.2 (Prototype) to evaluate their susceptibility to confounding factors, misclassification bias, selection bias, attrition bias, reporting bias and analysis bias. Studies deemed to be high risk based on critical appraisal results were excluded from further analysis. Descriptive data analysis was performed for studies considered low or unclear for risk of bias. Meta-analyses were conducted for antibiotic resistance and microbial diversity.

**Review findings:**

Out of 8370 screened records, 50 articles fulfilled the search criteria, and from these, 13 studies were included in meta-analysis. Most studies investigated the impact of aminoglycoside exposure on soil microbiota (93%) in a laboratory setting (62%), primarily from the United States (32%), China (24%), France, Switzerland and Germany (8%). A limited number of studies investigated the impact of aminoglycoside exposure on disease suppression, so it was excluded from meta-analysis. Therefore, our synthesis primarily details the impact of aminoglycoside exposure on the microbial diversity and antibiotic resistance of the soil microbiota. Overall, exposure to aminoglycosides did not result in a significant change in the microbial diversity. However, soil use, pH, and type of aminoglycoside used could be potential modifiers. Additionally, we observed an average 7% of the microbial population exhibiting resistance to aminoglycosides, with the relationship between the exposure concentration and the selection concentration emerging as a potential modifier.

**Conclusions:**

Current research is limited by gaps in understanding the relationship between aminoglycoside exposure, microbial community dynamics, and disease suppression, as well as by insufficient data on less-studied aminoglycosides and key confounding factors. Current research also suggests a potential relationship between antibiotic concentrations used for exposure and selection of resistant bacteria. These findings emphasize the need for informed antibiotic management policies and rigorous, targeted research to better understand the relationship between soil factors and antibiotic concentrations used on the impact of aminoglycosides on soil microbiota.

**Supplementary Information:**

The online version contains supplementary material available at 10.1186/s13750-025-00365-6.

## Background

Aminoglycosides are potent bactericidal antibiotics that inhibit protein synthesis [1]. Aminoglycosides were first isolated from soil bacteria, and some are frequently used in agricultural practices. Streptomycin, isolated from *Streptomyces griseus*, was the first aminoglycoside discovered in 1943. In addition to being the first isolated aminoglycoside, streptomycin is one of the three antibiotics currently registered by the EPA for use in plant protection in the United States [2, 3]. It is predominantly used in pear and apple orchards and more recently in citrus groves [4]. Other aminoglycosides have been discovered over time including neomycin (1949, *S. fradiae*), kanamycin (1957, *S. kanamyceticus*), gentamicin (1963, *Micromonospora purpurea*), kasugamycin (1965, *S. kasugaensis*), tobramycin (1965, *S. tenebrarius*), apramycin (1967, *S. tenebrarius*), and sisomicin (1976, *Micromonospora inyoensis*). With the emergence and spread of streptomycin-resistant strains of the pathogen *Erwinia amylovora* in pear and apple orchards, there is increased adoption of kasugamycin as an alternative to streptomycin [3, 5]. Aside from streptomycin and kasugamycin that are used in many countries around the world, gentamicin is also routinely used Mexico and Central America to control *Erwinia amylovora* in apple and pear as well as other diseases in vegetable crops caused by pathogenic *Pectobacterium*, *Pseudomonas*, *Ralstonia*, and *Xanthomonas* [3, 5].

In addition, to direct application of antibiotics in plant protection, the use of manure for fertilization can serve as a route of antibiotic exposure in soil. Aminoglycosides are used to increase meat production by preventing infections or outbreaks in livestock [6, 7]. The most used aminoglycosides in veterinary medicine are neomycin, dihydrostreptomycin, apramycin, gentamicin, kanamycin, paromomycin and neomycin [7]. In general, aminoglycosides are poorly absorbed in the gastrointestinal tract of animals and when injected, they are rapidly excreted in urine without metabolic transformation [8]. Thus, manure from antibiotic-treated animals can be a significant source of aminoglycoside exposure in the soil environment. Reported concentrations of aminoglycosides in manure range from an average of 1863 µg/kg of neomycin [9] to 558.37 µg/kg for neomycin, 284.15 µg/kg for gentamicin and 40.33 µg/kg for kanamycin [10]. Manure would not only bring antibiotics, but also a different population of bacteria that have been selected by the treatment and often harbor antibiotic resistance genes [8, 11]. Therefore, the impact of manure on soil bacteria might involve more complex dynamics than simple exposure to antibiotics. Other indirect routes of antibiotic exposure in agriculture include waste from humans excreted into water and soil through municipal wastewater, sewage sludge, and biosolid [11]. Among aminoglycosides, gentamicin, tobramycin, and amikacin would be the antibiotics of concern as they are the most used in humans [7, 8, 12]. Despite the high likelihood of aminoglycoside exposure in agriculture, there is very little research on the relationship between aminoglycoside exposure and the soil microbiota. Likewise, the potential role of these antibiotics in the soil environment and in plant health is not well understood.

Adsorption and degradation are some of the most important factors influencing the effects of antibiotics in the soil environment [13]. Aminoglycosides strongly adsorb to soil particles, especially clay and organic matter, due to their positive charges [14–16]. This adsorption is not only dependent on soil texture but also modulated by pH and temperature. Strong adsorption of aminoglycosides limits their bioavailability and thus their effect on the soil microbiota. However, it allows them to persist in the soil environment, which may potentially cause long term effects.

The microbial composition of the soil and roots plays a crucial role in maintaining the health of plants. Microorganisms are involved in organic matter turnover, nutrient release, stabilization of the soil structure, and soil fertility [17–19]. Moreover, microorganisms assist in nitrogen fixation [18] growth promotion [19], and stress tolerance in plants [20]. The microbiome associated with plant roots represents a distinct subset of the soil microbiome [21–23]. This is largely attributed to the strong selective environment created by the host plant to assemble a particular guild of bacteria [22–24]. Those found in the direct vicinity of the roots represent the rhizospheric bacteria while those colonizing internal plant tissues represent the endospheric bacteria. Given the bactericidal nature of aminoglycosides, they can inhibit the growth of microorganisms and thus influence the composition of the soil and root microbiomes. Understanding aminoglycoside-induced alterations in the soil and plant microbiota could provide insight on the effect of aminoglycoside exposure and plant health.

In bacteria, aminoglycoside resistance is well documented and takes many different forms including enzymatic modification [25], target site modification via an enzyme or chromosomal mutation [26–28], and efflux [29–34]. Aminoglycoside resistance was also discovered in the model plant *Arabidopsis thaliana.* It is associated with an ATP binding cassette (ABC) transporter, WBC19 (ABCG19), capable of conferring resistance to kanamycin [35], neomycin, geneticin, and paromomycin [36]. Exposure to aminoglycosides therefore may provide a selective advantage to microorganisms harboring aminoglycoside resistance genes that can then become dominant in the microbial or plant community. More broadly, the existence and evolution of such resistance genes suggest that plant and soil bacteria exposure to aminoglycosides is prevalent.

Pathogen-suppressive soils have a microbial composition that promotes overall plant health. This might involve the creation of conditions that prevent the establishment of pathogens, allowing the establishment of pathogens but pathogens fail to cause disease, or allowing the establishment of pathogens that can cause disease but disease severity declines with the continued monoculture of the host crop [20, 37, 38]. Aminoglycoside exposure has the potential to impact soil suppressiveness. For example, *Pseudomonas* species were shown to play a major role in suppressing the pathogens *Penicillium digitatum and P. italicum* by producing the antifungal compound diacetylphloroglucinol (DAPG) [39, 40]. Clinical evidence also shows that *Pseudomonas* sp. are susceptible to aminoglycosides [41]. Aminoglycoside exposure, therefore, has the potential to impact plant microbial composition that could lead to reduction of soil suppressive bacteria.

This study aims to address the above-mentioned knowledge gaps on how the soil and plant root associated microbiota are impacted by aminoglycoside exposure. We have systematically reviewed peer-reviewed literature and grey literature on academic and specialist organization databases. Using the evidence identified we provide a meta-analysis of aminoglycoside exposure impacts on microbial diversity and antibiotic resistance. The analysis also provides a novel insight into the possible impact of climate change on the soil microbiota.

### Stakeholder engagement

Local conventional and organic farmers were considered external stakeholders and consulted during the writing of the protocol [42]. Comments from stakeholders were taken into consideration and implemented into revisions of the protocol. Following completion of the review, we took stakeholder comments on accessibility of research findings into consideration and made our results available to the public and upon request.

### Objective

The objective of this systematic review was to collate existing research on the impact of aminoglycoside exposure on the soil and plant root-associated microbiome. The review also ascertains any knowledge gaps for future primary research areas.

### Primary question

What is the impact of aminoglycosides on the soil and plant root-associated microbiome composition?

### Secondary questions

Are aminoglycoside resistance genes enriched by aminoglycoside exposure?

Are soil pathogen suppressive bacteria reduced by aminoglycoside exposure?

Components of the question.

Population: Soil and plant root-associated microbiomes.

Exposure: aminoglycosides antibiotics.

Comparator: Control with no exposure (i.e., no aminoglycoside).

Outcome: Changes in overall species richness and diversity (microbial composition), changes of the resistome (i.e. the quantification of resistance genes), and the ability to suppress plant pathogens (e.g., Biomass reported as mg/kg; changes in banding patterns, or richness expressed as H’ and S’ indices, and abundance of resistance genes or suppressive pathogens reported as percentages).

## Methods

### Deviation from the protocol

In conducting our meta-analysis, we made several deviations from our initial protocol. Notably, we modified our database strategy by including only the first 50 articles from Web of Science and excluding the EPA database. The EPA database yielded a handful of hits that were not relevant. One the other hand Web of Science searches yielded an excessive number of hits (over 28,000) and thus the first 50 most relevant ones were retrieved. These were often found to overlap with those retrieved via other databases. Additionally, we adjusted our variables: we incorporated research focusing on soil use, replaced the soil type variable with reported continuous measures of percentage sand, silt, and clay, and utilized categorical data for plant species. Furthermore, when ranges were reported in the studies, we opted to use the maximum value for our analyses. These adjustments were made to enhance the relevance and applicability of our findings.

### Searching for articles

The protocol for this review was published by Coates et al. 2022 [42]. In the protocol, methods and criteria for relevance and validity were described prior to the study, in accordance with guidelines from the Collaboration for Environmental Evidence. The reporting of this systematic review also followed the ROSES reporting standards (see Additional file 5). The search aimed to retrieve a wide range of quantitative scientific evidence, i.e., research articles, conference presentations, or gray literature from specific websites covering the topic of aminoglycoside exposure and its impact on the soil and plant root-associated microbiome.

### Search terms and language

Some of the top consumers of aminoglycosides in agriculture are non-English speaking countries. To avoid eliminating articles that may include relevant information, we did not exclude articles based on language. We utilized translation tools, assistance from the university librarian, and help from native academic speakers when available to translate any articles not written in English. Any articles that could not be translated were excluded from our analysis. To ensure we were assembling the most up-to-date information, the search was last performed on September 29, 2022, or 6 months prior to the time of article submission. The search terms/keywords that were used to search for relevant literature were broken into two components: the population and the exposure and were combined using Boolean operators “AND” and/or “OR”. To ensure a comprehensive search that yielded at least 500 articles including benchmark articles, we excluded the comparator and outcome from our search term combinations. Benchmark articles identified during the scoping are described in our protocol [42].

Population terms: soil, plant, root, endosphere, rhizosphere, microbiome, microorganism, bacteria.

Exposure terms: aminoglycoside, kanamycin, streptomycin, gentamicin, neomycin, tobramycin, kasugamycin, amikacin, dihydrostreptomycin, apramycin, paromomycin.

### Databases

The search was conducted using these academic and non-academic databases:

1. Science Direct.
2. Scopus.
3. PubMed.
4. Google Scholar (first five pages).
5. Web of Science (Core Collection database; first five pages).

The final search string was:

(“soil” OR “endosphere” OR “rhizosphere” OR “plant” OR “root”) AND (“microbiome” OR “microorganism” OR “bacteria”) AND (“aminoglycoside” OR “kanamycin” OR “gentamicin” OR “neomycin” OR “streptomycin” OR “tobramycin” OR “amikacin” OR “dihydrostreptomycin” OR “apramycin” OR “paromomycin”).

Note - when going to retrieve 2022–2024 articles, we limited the subject area to Agricultural and Biological Sciences and Environmental Science; for publication stage, both articles in press and final were selected, also limited document type to articles only.

Specific antibiotic names were excluded from the ScienceDirect search to adhere to boolean key term limits. Only articles in the press were included from the Scopus search. Any articles still under review were excluded from the Scopus search. Our search terms for the academic databases retrieved all 15 benchmark articles.

### Specialist searches

The following search strategy was also conducted using the USDA (U.S. Department of Agriculture) PubAg website:

(soil AND bacteria) AND (aminoglycoside OR kanamycin OR gentamicin OR neomycin OR streptomycinOR tobramycin OR amikacin OR dihydrostreptomycin OR apramycin OR paromomycin).

### Supplementary searches

JC identified additional relevant articles by forward citation, a review of articles that cite relevant literature, and backward citation, articles cited by relevant literature, tracing on all articles included after the full-text screening.

### Article screening and study eligibility criteria

#### Screening process

Search results from each academic and internet database were exported to the Covidence systematic review management software. The management software removed any duplicates present and manual curation was used to remove any additional duplicates discovered. Two reviewers (JC and KB) independently screened the title and abstracts of exported articles. Any disagreements or ambiguity was identified through the Covidence software and resolved by review from a senior third party (MA) prior to full-text screening. Reviewers (JC and KB) independently completed a second level of screening to review the full text of the titles and abstracts meeting the eligibility criteria. Any disagreements or ambiguity were identified through the Covidence software and resolved by review from a senior third party (MA). Records of the number of articles excluded and reasons for their exclusion, at full-text level, are provided as an Additional File.

#### Eligibility criteria

For a study to be included in the systematic review, it must have met the following criteria:

Eligible population: plant and root-associated microbiota: soil, endosphere, rhizosphere, or root.

Eligible exposure: aminoglycosides: kanamycin, gentamicin, neomycin, tobramycin, streptomycin, kasugamycin, amikacin, dihydrostreptomycin, apramycin, paromomycin.

Eligible comparator: no aminoglycoside.

Eligible outcome: Changes in overall species richness and diversity (microbial composition, richness expressed as H’ and S’ indices), change of the resistome (i.e. relative abundance of resistance genes, percent of surviving bacteria in the presence of antibiotic), and the ability to suppress plant pathogens (e.g., suppression of pathogens reported as percentages).

Eligible study types: Both field and laboratory control-interventions studies that include aminoglycoside exposure and show comparison to comparator i.e., no aminoglycoside application to soil.

Eligible types of articles: peer-reviewed research articles, conference presentations, other grey literature from the specified websites.

### Study validity assessment

Studies deemed eligible by the previously mentioned criteria were subjected to critical appraisal by two reviewers (JC and AL) during the full-text screening. The CEE Critical Appraisal Tool Version 0.2 (Prototype) [43], was modified to our review question, and used for study validity. The CEE Critical Appraisal Tool was used to assess and categorize each study’s susceptibility to confounding factors, misclassification bias, selection bias, attrition bias, reporting bias and analysis bias. Any disagreements were resolved by a senior level reviewer (MA). For procedural independence, none of the reviewers reviewed any articles they authored. Articles deemed as high risk for bias in any category were excluded from further downstream analysis.

### Data coding and extraction strategy

Using Covidence, evidence tables of meta-data and data extraction (i.e., study findings) were produced. For each screened study that fit the inclusion criteria and met the study validity criteria, data was extracted according to predetermined codes described in the protocol [42]. The following data was coded for:

*Bibliographic information (author, year, title, source of publication).

*Study location (country).

*Study site (feld/laboratory).

*Population (Soil, root, endosphere, rhizosphere).

*Soil type (% sandy, %clay, %silt).

*Soil use (agricultural, natural, research).

*Type of tillage (no-till, deep (deeper than 10 inches), medium-depth (5–10 inches), shallow (1–4 inches)).

*Plant species (name).

*Experimental conditions (light vs. dark, temperature, duration).

*Soil properties (pH, organic matter content, moisture, oxygen status, compost use).

*Antibiotic characteristics (name, chemical formula, concentration, method of application, frequency of application, previous history of antibiotic exposure, and limits of detection).

*Method for evaluating microbial composition, resistance genes, and soil suppressiveness (e.g., Phospholipid Fatty Acid Analysis (PFLA), Terminal Restriction Fragment Length Polymorphism (T-RFLP), Denaturing Gradient Gel Electrophoresis (DGGE), Shotgun Sequencing, High throughput sequencing, 16 S rRNA gene sequencing).

*Reported mean and standard deviation of microbial composition, resistance genes, and soil pathogen suppression for the control and intervention/experimental groups (e.g., Biomass reported as mg/kg; changes in banding patterns, or richness expressed as H’ and S’ indices, and abundance of resistance genes or suppressive pathogens reported as percentages).

*Comparator (description of the control with no exposure, i.e. no aminoglycosides).

Two reviewers (JC and AL) simultaneously and independently extracted data from all studies deemed eligible based on the inclusion criteria. Any disagreements between the two reviewers were resolved by a senior level third reviewer (MA). If relevant data was either missing or ambiguous, the corresponding authors of those studies were contacted. In the event of no response, we indicated no response in our metadata and only reported available data in our final analysis. If the amount of missing data is deemed to be substantial (> 50%), the study or variable was excluded from our final analysis.

### Potential effect modifiers/reasons for heterogeneity

The identified potential effect modifiers and sources for heterogeneity are listed below. Also mentioned are the methods of testing.

*Soil type (i.e. sandy, loamy, clay, silt) [subgroup analysis].

*Soil usage (agricultural, natural) [subgroup analysis].

*Type of tillage (no-till, deep (deeper than 10 inches), medium-depth (5–10 inches), shallow (1–4 inches)) [subgroup analysis].

*Plant species [subgroup analysis].

*Experimental conditions (light vs. dark), the method used [subgroup analysis], and temperature and duration in days [meta-regression].

*Properties of the soil (pH, organic matter content, moisture) [meta-regression] and (oxygen content status, compost use) [subgroup analysis].

*Antibiotic characteristics (name), method of application, previous exposure of antibiotics [subgroup analysis] and concentration, half-life, sorption coefficient, frequency of application [meta-regression].

This list was developed based on a preliminary literature search performed by KB, BJ, and NC with input from JC. MA provided overall scientific expertise.

### Data synthesis and presentation

After data extraction from all eligible studies, a narrative descriptive synthesis was conducted on those demonstrating a low and medium risk of bias (based on study validity assessment), summarizing information in tables and figures. Data was assembled utilizing Microsoft Excel. Summaries were descriptive, outlining bibliographic information, study location, and site, population, soil characteristics, aminoglycoside characteristics, and experimental methods and conditions.

To assess the risk of publication bias, effects from individual studies were visualized in funnel plots. Where evidence allowed, efforts were made to estimate the effect size of the outcome (i.e. microbial composition, antibiotic resistance genes, and soil suppression after aminoglycoside exposure relative to the comparator).

### Quantitative synthesis

#### Review findings

When data was appropriate for quantitative synthesis (e.g. meta-analysis), we aimed to calculate the effect size. The standardized mean differences of the Shannon and Simpson Diversity Indices were estimated to determine the impact of aminoglycoside exposure on microbial diversity. The Resistance Quotient (RQ) was estimated to determine the impact of aminoglycoside exposure on antibiotic resistance. Due to the nature of ecological studies, multiple values were reported per study. Therefore, we opted to perform multi-level hierarchical meta analysis to estimate the overall effect size. Collinear variables, missing variables (less than 50% completeness), and variables with a near zero variance were excluded from the hierarchical modeling. i2 was calculated to evaluate the variance.

#### Review descriptive statistics

**Fig. 1:**
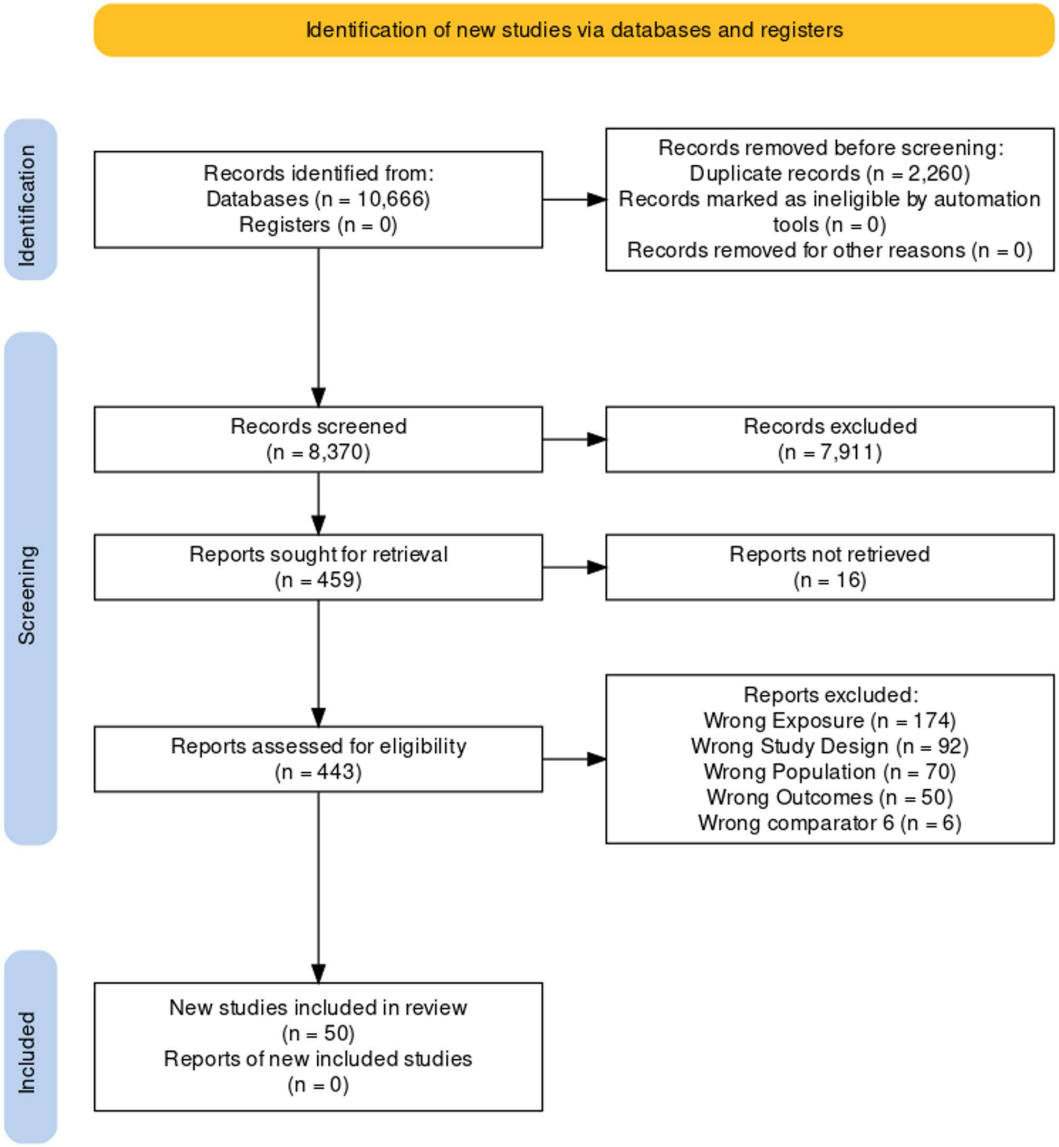
A flow diagram reporting all stages of the inclusion/exclusion process, from search results to full text eligibility

The initial searches yielded 8,370 unique records (Fig. 1). Four hundred fifty nine titles and abstracts were deemed relevant for full-text screening. Four hundred eight full-texts were excluded from further analysis after full-text screening. The most common reason for exclusion was due to studies investigating the wrong exposure (*n* = 174). Articles were also excluded due to wrong study design (*n* = 92), wrong outcomes (*n* = 50), wrong population (*n* = 70), no full-text available (*n* = 16), and wrong comparator (*n* = 6) (Fig. 1).

Fifty articles were evaluated for risk of bias. Twenty-four of the fifty articles evaluated for risk of bias were deemed to be high risk and were excluded from any downstream analysis. Excluded articles were most often found to have high risk for confounding factors (*n* = 17), misclassification bias (*n* = 9), reporting bias (*n* = 8), and analysis bias (*n* = 3). Ultimately, 26 studies were included in the narrative synthesis and were considered for quantitative analysis.

### Narrative synthesis

**Fig. 2:**
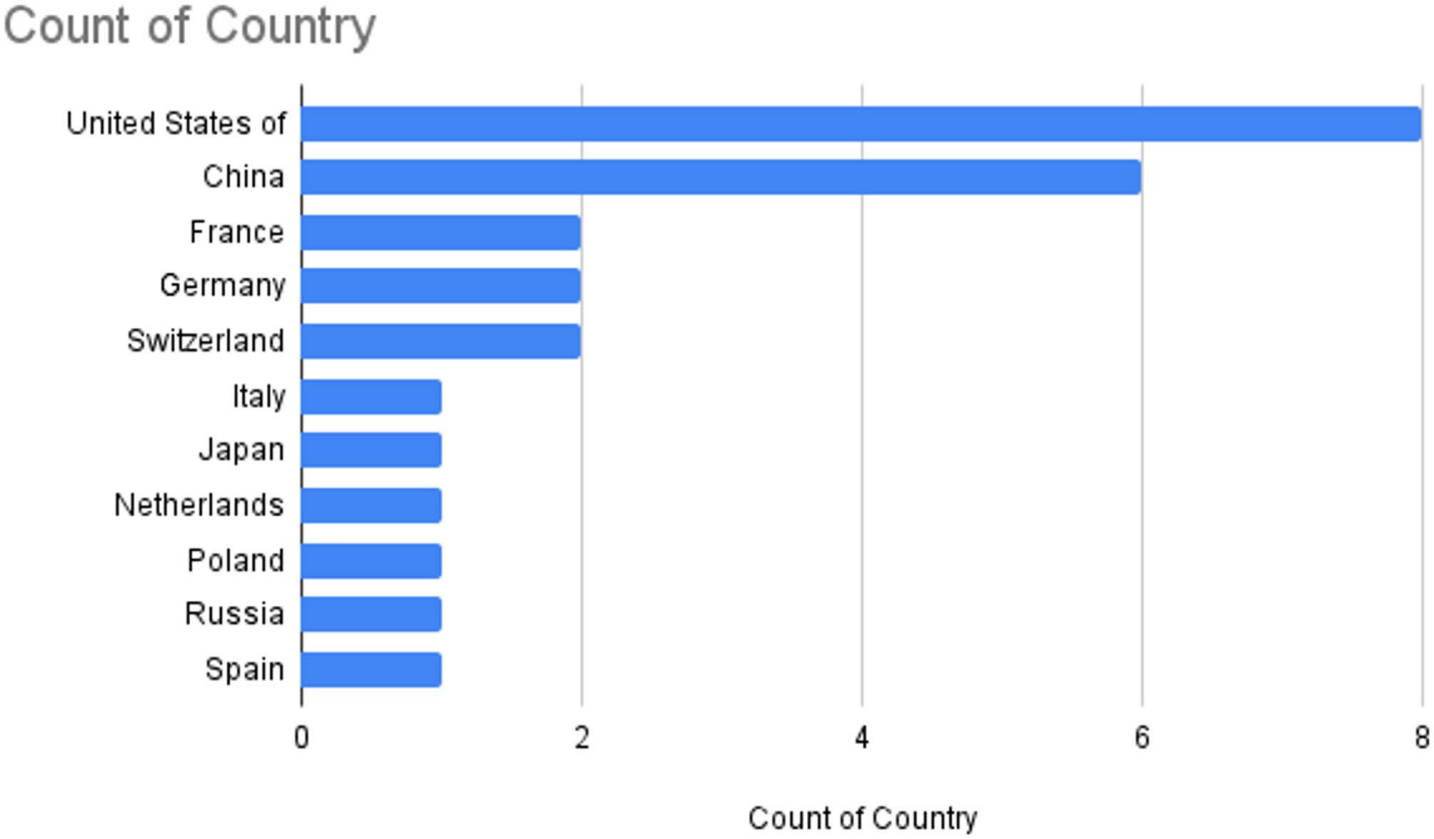
Bar chart of study locations investigating the impact of aminoglycoside exposure on the plant and root associated microbiota

Data was reported from 11 different countries across 3 continents. Samples were most often collected in the United States (*n* = 8; 31%), China (*n* = 6; 23%), France, Switzerland and Germany (*n* = 2; 8% each) (Fig. 2).The remaining samples were collected from European (Netherlands, Spain, Poland, and Italy: *n* = 1; 4% each), Asia (Japan and Russia: *n* = 1; 4% each).

**Fig. 3:**
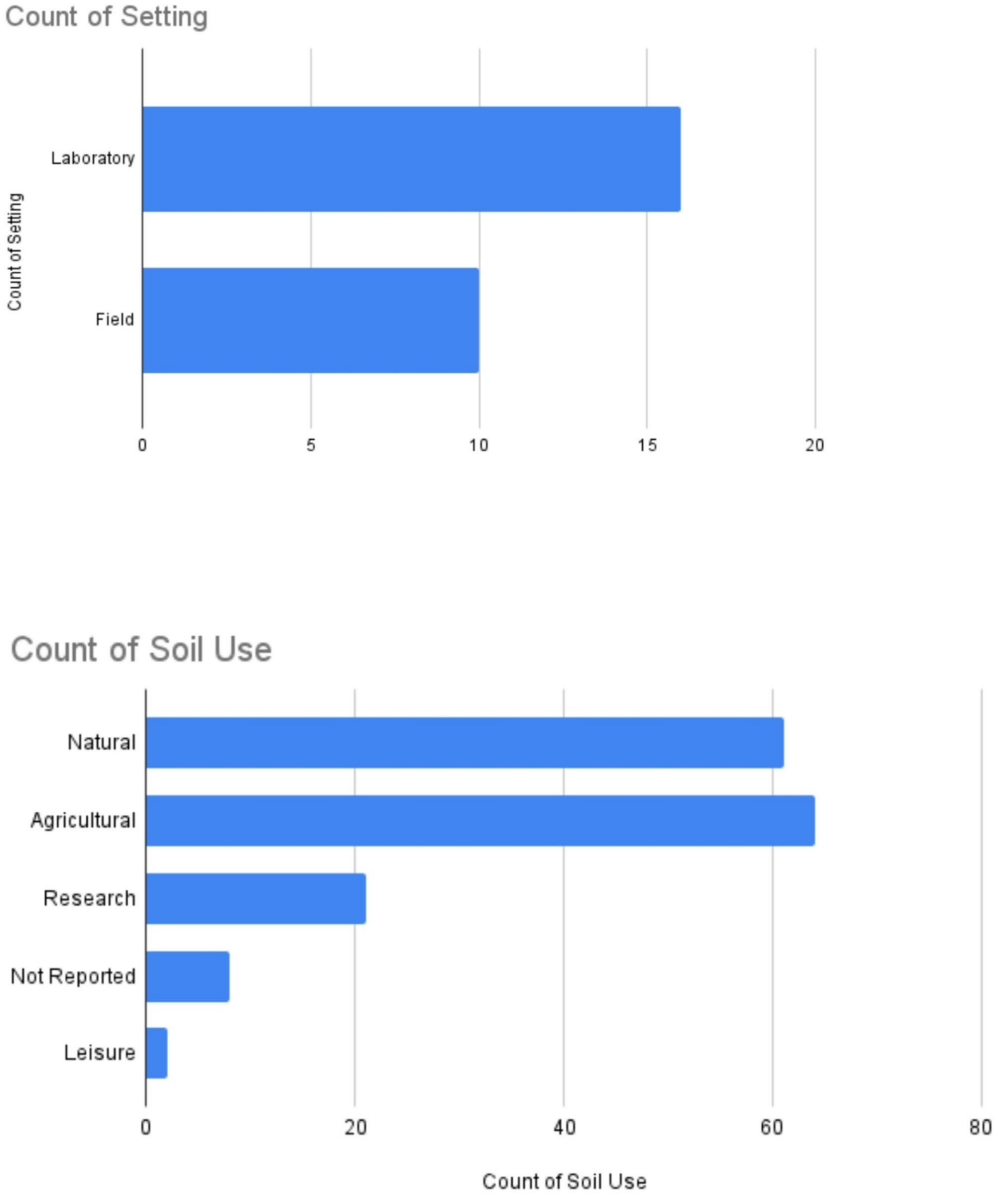
Barplots describing the (**A**) setting type and (**B**) soil use of included studies

There was a lower representation of studies where aminoglycoside exposure occurred in a field setting (39%) compared to studies where aminoglycoside exposure occurred in a laboratory setting (62%) (Fig. 3A). A total of 158 samples were investigated in the included studies (control: 39 (25%); exposed to aminoglycosides: 119 (75%)). Samples were primarily taken from the soil microbiome. However, 7% of samples were isolated from the rhizosphere. Prior soil use was not reported for 5% of the samples. When reported, soils were most often used for agricultural purposes (41%) or considered pristine natural soils (39%) prior to experimentation. Other soils were used for research studies (14%) or were used for leisure human activities (< 1%) (Fig. 3B).

The baseline plant species was unknown for 23.7% of the samples reported by the studies. However, when the baseline plant was described, the majority of the soils were used to grow angiosperms (*n* = 94; 77%) versus gymnosperms (*n* = 28; 23%).

**Fig. 4:**
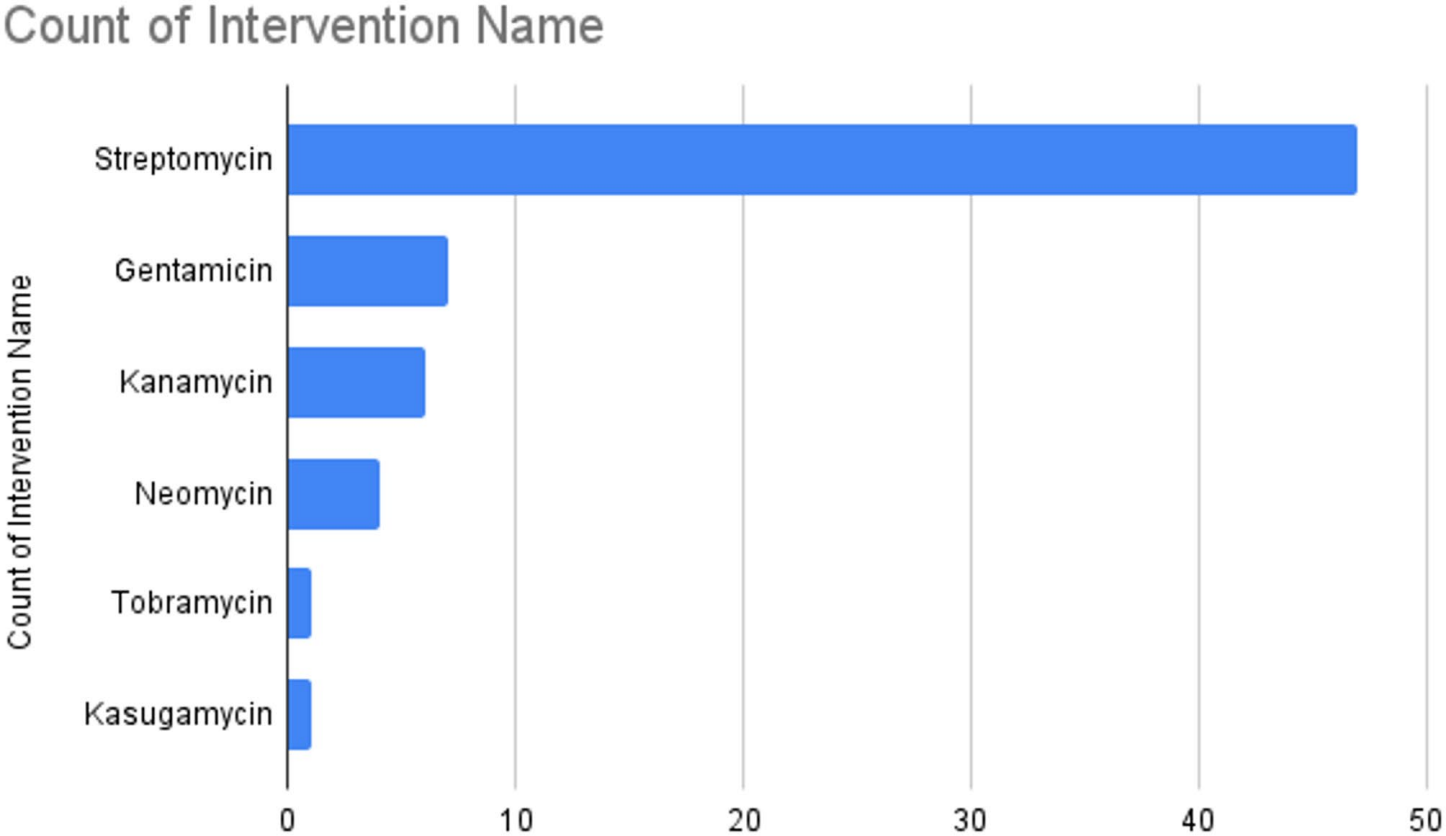
Bar chart of aminoglycoside interventions explored in studies. Note: N is greater than the number of studies because select studies investigated multiple interventions

The impact of exposure from a range of aminoglycosides was explored including streptomycin (*n* = 47; 71%), gentamicin (*n* = 7; 11%), kanamycin (*n* = 6; 9%), neomycin (*n* = 4; 6%), tobramycin (*n* = 1; 2%) and kasugamycin (*n* = 1; 2%) (Fig. 4). The concentrations of antibiotic soils were exposed to ranged from 1.0 × 10^-4 mg/g to 2.0 × 10^1 mg/g of soil. Water was the most commonly used control. Our search did not yield studies investigating the impact of amikacin, dihydrostreptomycin, apramycin, or paromomycin.

Less than 50% of the studies reported information about compost use, baseline nitrogen content, previous history of antibiotic exposure, baseline temperature, silt composition of soil, clay composition of soil, sand composition of soil, baseline carbon content, anaerobic vs. aerobic experimental conditions, type of tillage, and water holding capacity. Due to the lack of reporting for the aforementioned variables, they were excluded from downstream analysis thus leaving the question of their effect on the impact of aminoglycoside exposure on the plant and root associated microbiota inconclusive.

For full transparency, the following additional files are included:

Additional File 1: Database search results.

Additional File 2: Studies excluded at full-text with reasons for exclusion.

Additional File 3: References for studies included in the appraisal.

Additional File 4: Critical Appraisal results.

Additional File 5: Qualitative and quantitative data extraction from included studies.

### Data synthesis

#### Studies that reported microbial diversity

Of the 26 studies that met our eligibility criteria, 16 studies investigated the impact of aminoglycoside exposure on microbial diversity. Studies were conducted in European countries (*n* = 6; 38%), China (*n* = 6; 38%), the United States (3; 19%), and Russia (1; 6%). Primarily soil samples (*n* = 12; 75%) were collected. However, 4 studies obtained soil from the rhizosphere. There were no samples collected from the endosphere. Setting was defined for all included studies with investigation conducted in either a laboratory (*n* = 11; 69%) or field (*n* = 5; 31%). Soil use prior to sample collection was defined for 88% of the samples and included use for research (*n* = 3; 19%), agriculture (*n* = 7; 44%), leisure (*n* = 1; 6%) or natural (*n* = 3; 19%) purposes. The native plant species was not described for 38% of the studies. When described, all samples were taken from soils used to grow angiosperms. pH and the depth of collection differed among the samples. The mean soil pH in experiments was 6.8 but values ranged from 3.95 to 9.1. Soils on average were collected from a depth of 22 cm but values ranged from 4 to 60 cm. Included studies investigating the impact of aminoglycoside exposure on microbial diversity, solely examined the impact of streptomycin (*n* = 7; 78%), gentamicin (*n* = 1; 11%), and kanamycin (*n* = 1; 11%) applied directly to the soil (41.5%), manure applied to the soil (47.4%), or sprayed on plants (11.1%). No other aminoglycosides were investigated for their impact on microbial diversity. Seven methods were used to estimate the impact of aminoglycoside exposure on microbial composition including 16 S rRNA gene sequencing (*n* = 8; 50%), Dilution Plating (*n* = 3; 19%), Average Well Color Development (*n* = 1; 6%), Selective Inhibition/Substrate Induced Respiration (*n* = 1; 6%), Barcoded Pyrosequencing (*n* = 1; 6%), Denaturing gradient gel electrophoresis (*n* = 1; 6%) and Chloroform fumigation extraction (*n* = 1; 6%). To have a dataset of sufficient size for meta-analysis, we selected studies utilizing 16 S rRNA gene sequencing for comparison using meta-analysis. The 16 S rRNA gene sequencing results were estimated using multiple different metrics including: Shannon Diversity (*n* = 9), Simpson Diversity (*n* = 5), Chao1 Richness (*n* = 4), OTU Richness (*n* = 3), Ace (*n* = 1), and Tau (*n* = 1). Some papers measured multiple diversity metrics. Given the differences in these metrics, we opted not to combine the metrics and perform a meta-analysis. Therefore, our meta-analysis relied on studies that utilized 16 S rRNA gene sequencing to measure the Shannon or Simpson Diversity.

#### Microbial diversity: relationship between aminoglycoside exposure and the alpha diversity of bacterial populations

**Fig. 5:**
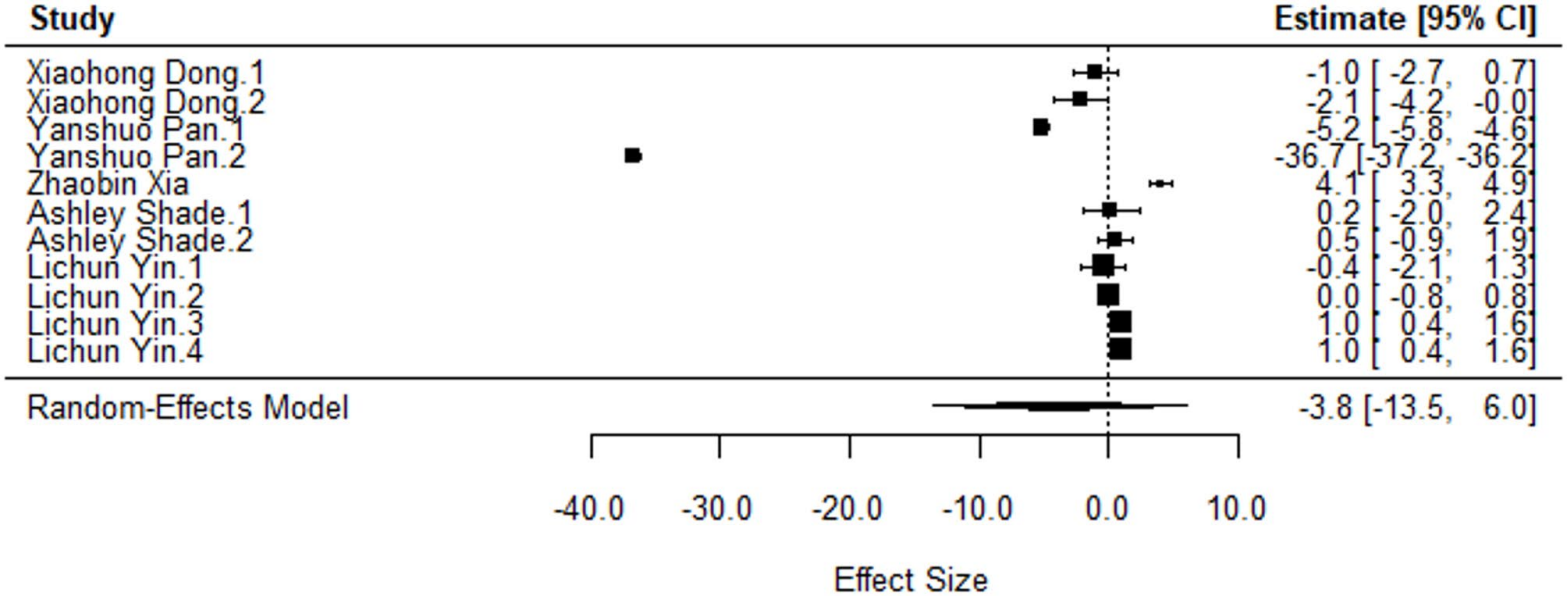
Standardized mean difference and 95% confidence intervals of articles investigating the impact of aminoglycoside exposure on the Shannon diversity

**Fig. 6:**
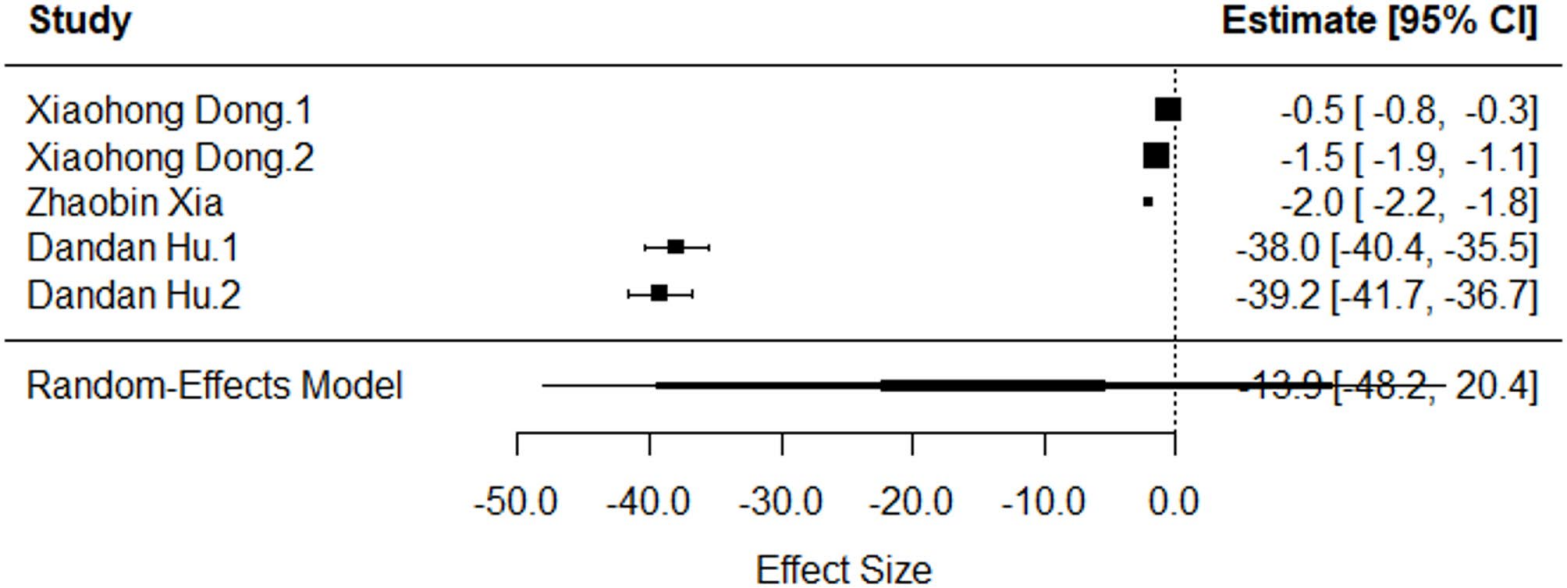
Standardized mean difference and 95% confidence intervals of articles investigating the impact of aminoglycoside exposure on the Simpson diversity

Eight studies meeting predefined criteria were included in this meta-analysis, demonstrating methodological comparability in estimating microbial diversity and quantifying it based on the Alpha diversity. To address challenges stemming from multiple antibiotic treatments and diverse soil populations within the studies, we opted to use multilevel hierarchical modeling to estimate the effect size. A restricted maximum-likelihood model with a and for the standardized mean difference was utilized to acknowledge inherent heterogeneity. Stratification by Alpha diversity measurement yielded two distinct groups: estimates measured using Shannon Diversity, and those measured using Simpson Diversity. Comparative analysis of the former revealed a non-significant difference in effect sizes (*p* = 0.41), with an overall effect size of -3.78 (95% CI: -13.54–5.98) (Fig. 5). Analysis of Simpson Diversity data resulted in a statistically non-significant distinction in effect size was observed (*p* = 0.33), with an overall effect size of -14.07 (95% CI: -48.96-0.82) (Fig. 6).

The most notable observation was the considerable heterogeneity present within the dataset. The between-study heterogeneity variance for the Shannon Diversity was estimated at τ2 level 2 = 83.62 and τ2 level 3 = 53.46. This means that I2 Level 2 = 60.92% of the total variation can be attributed to within-cluster heterogeneity, and I2 Level 3 = 38.95% to between-cluster variation. Quantifying this variability using the τ2 level 2 and τ2 level 3 metrics for Simpson diversity showcased substantial between-study heterogeneity contributing to our results, τ2 level 2 = 0.1% and τ2 level 3 = 99.89%.

Further investigation revealed three outliers contributing to the heterogeneity observed in the Shannon Diversity results. When excluded from the meta-analysis, the I2 level2 value reduced to 12.7% suggesting low to moderate heterogeneity within studies (Table 1). Therefore, the studies were excluded from further analysis. However, the standardized mean difference still remained non-significant and there was significant level3 variation suggesting another source of heterogeneity.

**Table 1:** Impact of influential cases on heterogeneity observed in Shannon diversity score

Analysis	SMD	95% CI	*p*	I2 level2	I2 level3
Main analysis	-3.78	[-13.54,5.98 ]	0.41	60.9%	39.0%
Leave-one-out^1^	-0.04	[-1.50,1.43]	0.96	12.7%	65.2%

^1^Removed: Yanshuo Pan 2019 and Zhaobin Xia 2023

One datapoint from the Dandan Hu 2019 study was identified as an outlier that contributed to the heterogeneity observed in the Simpson Diversity results. However, leave-one-out analysis demonstrated that Dandan Hu 2019 did not significantly influence the heterogeneity observed in the results for Simpson diversity. When excluded from the meta-analysis, there was no reduction in the I2 level value suggesting another contributing factor to the between-study heterogeneity and therefore was not excluded from downstream analysis (Table 2).

**Table 2:** Impact of influential cases on heterogeneity observed in Simpson diversity score

Analysis	SMD	95% CI	*p*	I2 level3
Main analysis	-4.63	[-17.85;8.99]	0.29	97.90%
Leave-one-out^1^	-20.09	[-80.92,40.74]	0.37	99.90%

^1^Removed: Dandan Hu 2019

Given the large between-cluster variance observed for the Shannon Diversity when removing the outliers, we conducted subgroup analyses to investigate the relationship between the categorical and continuous variables that varied between the studies: method of administration, nitrogen content, carbon content, risk of bias, native plant species, country, setting of exposure, type of intervention, pH of soil, soil use, year of publication, and duration of exposure. Moderators were analyzed using subgroup analyses, with pooled effect sizes computed separately for each subgroup. Most variables did not have a significant impact on the SMD for Shannon Diversity. However, the intervention type, soil pH, and soil use did have a significant impact (*p* < 0.005) on the SMD. Exposure to streptomycin SMD = 0.08 (95% CI: -0.07-0.03; *p* = 0.04), neutral soil SMD = 0.59 (95% CI: 0.19–3.93; *p* = 0.04), and soil used for agriculture SMD = 0.59 (95% CI: -0.01-1.20; *p* = 0.05) led to a significantly higher difference in the Shannon Diversity (Table 3).

**Table 3:** Subgroup analysis for Shannon diversity standardized mean difference

Subgroup	SMD	95% CI	*p*	*p* (subgroup)
Intervention				0.04
Kanamycin	-1.47	[-3.24,0.30]	0.09	
Streptomycin	0.08	[-0.07,0.03]	0.04	
Method				0.12
Illumina MiSeq	-1.47	[-4.47,0.72]	0.12	
ITS Sequencing	0.61	[-1.73,2.14]	0.80	
Continuous Metrics				
Nitrogen Content	-1.51	[-4.12,0.72]	0.08	0.08
Year	-647.13	[-2.81,3.44]	0.82	0.82
Carbon Content	-1.58	[-0.0012,0.02]	0.08	0.08
Duration	-5.58	[-0.13,0.20]	0.64	0.64
Risk of Bias				0.48
Low	-0.51	[-2.8,1.8]	0.61	
Unclear	0.61	[-2.5,4.8]	0.48	
Setting				0.71
Field	0.41	[-3.3,4.1]	0.80	
Laboratory	-0.32	[-5.21,3.78]	0.71	
pH				0.04
Alkaline	-1.47	[-3.24,0.3]	0.09	
Neutral	0.59	[0.19,3.93]	0.04	
Soil Use				0.04
Agriculture	0.59	[-0.01,1.20]	0.05	
Natural	-1.47	[-3.93,0.19]	0.04	

To further investigate the between-study heterogeneity observed when calculating the Simpson Diversity, we performed moderator analyses to investigate the relationship between the categorical and continuous variables that varied between the studies: method of administration, risk of bias, soil type, type of intervention, pH of soil, soil use, year of publication, and duration of exposure. Moderators were analyzed using subgroup analyses, with pooled effect sizes computed separately for each subgroup. Most variables did not have a significant impact on the SMD for Simpson Diversity. However, the method of exposure, type of intervention, and soil use did have a significant impact (*p* < 0.005) on the SMD. The use of spray to apply antibiotics SMD = -39.21 (95% CI: -41.68,-33.94; *p* < 0.001), exposure to streptomycin SMD = -39.21 (95% CI: -43.87- -32.59; *p* = 0.001), and soil primarily used for research SMD = -39.22 (95% CI:-43.18,-31.17; *p* = 0.001) led to a significantly lower Simpson Diversity (Table 4).

**Table 4:** Subgroup analysis for Simpson diversity standardized mean difference

Subgroup	SMD	95% CI	*p*	*p* (subgroup)
Method				< 0.001
Soil	-1.40	[-3.04,0.24]	0.07	
Spray	-39.21	[-41.68,-33.94]	< 0.001	
Intervention				0.002
Kanamycin	-0.99	[-3.9,1.97]	0.29	
Streptomycin	-39.21	[-43.87,-32.59]	0.001	
Tobramycin	-2.04	[-5.72,3.61]	0.43	
Continuous Metrics				
Year	-647.13	[-2.81,3.44]	0.82	0.82
Duration	-5.58	[-0.13,0.20]	0.64	0.64
Risk of Bias				0.62
Low	-20.09	[-80.92,40.74]	0.37	
Unclear	-2.04	[-2.5,4.8]	0.62	
pH				0.62
Alkaline	-20.09	[-123.41,87.31]	0.62	
Neutral	-2.04	[-88.06,83.98]	0.94	
Soil Use				0.04
Leisure	-2.04	[-5.64,1.57]	0.14	
Natural	-0.99	[-3.61,5.72]	0.43	
Research	-39.22	[-43.18,-31.17]	0.001	
Soil Type				0.59
Clay Loam	-0.99	-84.61-82.6 4	0.97	
Sandy	-20.62	-122.07-82.80	0.59	

### Studies that reported antibiotic resistance

Of the studies included in our analysis, 12 studies investigated the impact of aminoglycoside exposure on antibiotic resistance in the soil. Most studies were conducted using soil (*n* = 112; 92%) while only one study aimed to collect rhizosphere soil. Samples were most often collected from the United States (*n* = 4). The remaining samples were collected from Europe (Germany, Poland, Switzerland, France; *n* = 8), and China (*n* = 2). Most soil samples (54%) were exposed to aminoglycosides in field settings instead of in laboratory controlled settings. Soil samples were most often used for agricultural purposes (69%) compared to natural uses (8%). However, soil use was not reported for 24% of the samples. Plant species were not reported for 44% of the samples but when reported 36% of the samples were from fruits (Apple: 18%, Pear: 18%), vegetables (23%), or a pine tree (11%). Soil characteristics such as pH and depth of sampling in centimeters varied between samples. The mean pH of soil samples was slightly acidic at 6.5 but ranged from acidic to basic (4.7 to 7.8). Soil samples on average were collected from a depth of 14.3 cm but values ranged from 5.0 to 20.0 cm. The impact of streptomycin exposure on antibiotic resistance was investigated in 71% of the samples. Other aminoglycosides investigated included neomycin (11%), gentamicin (11%), kasugamycin (3%) and kanamycin (5%). The remaining samples were considered controls. Studies used different methods for describing controls including water (23%) or manure treated soil (8%). The method of aminoglycoside exposure was reported for 84% of studies. Populations were directly exposed to aminoglycosides through spraying methods for field studies (16%), directly to the soil in field studies (18%), to the soil through manure application (26%), or directly to the soil in microcosm laboratory studies (23%). Antibiotic resistance was most commonly determined using dilution plating (34%) to estimate the number of antibiotic resistant bacteria, or resistance quotient (RQ). Other methods for determining antibiotic resistance included OD_595_ (6%), and qPCR (45%) to quantify aadA, strA, tetW, tetM, and tetQ in samples and thus were excluded from the meta-analysis.

### Antibiotic resistance: relationship between aminoglycoside exposure and the resistance quotient

**Fig. 7:**
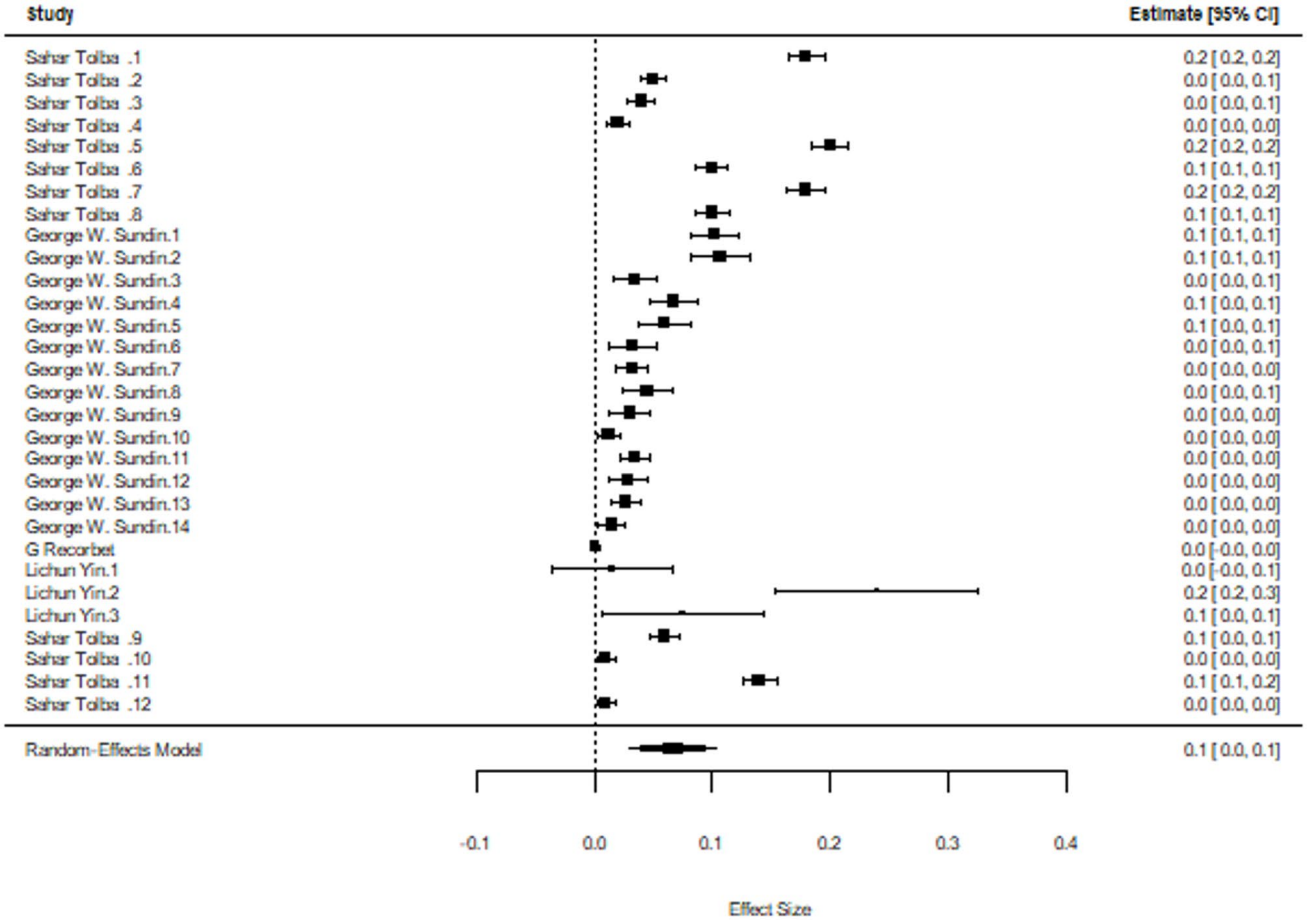
Proportion of viable cells following exposure to aminoglycosides

Five studies meeting predefined criteria were included in this meta-analysis, demonstrating methodological comparability in estimating antibiotic resistance and quantifying it based on the resistance quotient which is defined as the proportion of resistant isolates derived from the ratio of the viable count. To address challenges stemming from a unit of analysis problem, we opted to utilize a three-level meta analytic model. The pooled RQ value was RQ = 0.07 (95% CI: 0.03–0.10; *p* < 0.05) (Fig. 7). The estimated variance components were τ2 level 2 = 0.003 and τ2 level 3 = 0.0006. This means that I2 Level 2 = 82.9% of the total variation can be attributed to within-cluster heterogeneity, and I2 Level 3 = 16.0% to between-cluster variation. We found that the three-level model did not provide a significantly better fit compared to a two-level model with level three heterogeneity constrained to zero (𝝌_1_^2^=1.5, *p* = 0.2).

Influential analysis demonstrated that Lichun Yin 2023 influenced the effect size observed in the results for the RQ. However, when excluded from the meta-analysis, there was only a partial reduction in the I^2 value suggesting there was another factor contributing to the heterogeneity (Table 5).

**Table 5:** Impact of influential cases on heterogeneity observed in resistance quotient

Analysis	proportion	95% CI	*p*	I2 Level 2
Main analysis	0.07	[0.03,0.10]	< 0.05	82.90%
Leave-one-out^1^	0.06	[0.02,0.09]	< 0.005	79.50%

^1^Excluded: Linchun Yin 2023 Control

Given the large within-cluster variance observed, we conducted moderator analyses to investigate the relationship between the selection concentration, the type of intervention, the duration of exposure and the RQ value. When the selection concentration was below the exposure concentration, the concentration type was defined as sub. If the selection concentration was greater than the exposure concentration then the concentration type was deemed super. Categorical moderators were analyzed using subgroup analyses, with pooled effect sizes computed separately for each subgroup. Intervention type and duration in days did not have a significant impact on RQ. However, the selection concentration used to estimate the RQ did have a significant impact (*p* < 0.005). Selection concentrations that were lower than the exposure concentration also had significantly higher RQ values 0.14 (95% CI: 0.05–0.16; *p* < 0.0001) (Table 6).

**Table 6:** Subgroup analysis of the relationship between within-study variables and the resistance quotient

Subgroup	proportion	95% CI	*p*	*p* (subgroup)
Intervention				0.58
Control	0.07	0.01–0.12	0.01	
Kanamycin	0.001	[-0.19,0.06]	0.3	
Streptomycin	0.06	[-0.05,0.04]	0.87	
Concentration Type				0.002
Sub	0.14	0.05–0.16	0.0006	
Super	0.05	-0.09	0.05	
Duration (days)				0.94
Days	0.06	-0.1002	0.94	

### Studies that reported disease suppression

Three of the included studies investigated the impact of aminoglycoside exposure on disease suppression. Two studies were conducted using samples from the United States and used dilution plating to measure the impact of streptomycin exposure on disease incidence of *Erwinia herbicola* or *Monosporascus cannonballus*. The remaining study utilized soil from Japan and used qPCR to investigate the effect of streptomycin on suppression of Soybean Cyst Nematode *Heterodera glycines*. Given the small sample size, a meta-analysis was not conducted on the impact of aminoglycoside exposure on disease suppression. Therefore, the answer to the question of the impact of aminoglycoside exposure on disease suppression was inconclusive.

### Review limitations

In conducting this systematic review, we encountered several limitations that impacted the breadth and depth of our study’s findings. Our search strategy, although designed to be thorough and exhaustive, unfortunately did not yield a substantial number of research articles focused on assessing the impact of aminoglycoside exposure on disease suppression in soil ecosystems. This limitation inherently restricted our ability to comprehensively measure and analyze the effects of aminoglycoside exposure on disease suppression. The scarcity of studies directly exploring this relationship underscores the need for further research in this area to build a more robust understanding.

Another notable limitation lies in the exclusion of specific aminoglycosides from our analysis. Our search failed to identify studies investigating the effects of tobramycin, amikacin, dihydrostreptomycin, apramycin, and paromomycin on the plant and root-associated microbiota likely because these antibiotics are not commonly used in agricultural practices. This omission introduces a potential bias, as these excluded aminoglycosides might exhibit different impacts on soil microbial communities and disease suppression dynamics. Additionally, the lack of studies exploring the impact of aminoglycoside exposure within the endosphere, a crucial ecological niche, limits our ability to provide a comprehensive overview of the impact of aminoglycosides specifically on the plant root.

It’s important to note that data quality played a significant role in shaping our analysis. Several studies were excluded due to issues with study design, such as an inability to distinctly define control groups with no aminoglycoside exposure or incomplete reporting of data that directly impacted our ability to interpret outcomes accurately. Moreover, less than half of the studies provided information on critical confounding factors, such as compost usage, baseline nitrogen content, history of antibiotic exposure, soil composition, and other variables that significantly influence microbial diversity and antibiotic efficacy. The absence of this data limited our capacity to account for potential confounding effects in our analysis.

While our systematic review followed a robust methodology, these limitations underscore the complexity of studying the relationship between aminoglycoside exposure and disease suppression in soil ecosystems. Addressing these limitations in future research endeavors will contribute to a more comprehensive understanding of the intricate interplay between aminoglycosides, microbial communities, and disease dynamics in soil ecosystems.

### Review conclusions

Exposure to aminoglycosides has minimal impact on the alpha diversity of soil microbial communities, with no statistically significant changes observed. However, variations in pH, soil use and aminoglycoside used suggest a potential influence on diversity that warrants further exploration. Additionally, exposure to aminoglycosides leads to a small proportion of resistant bacteria, averaging less than 10%, with the relationship between the exposure concentration and the concentration defined as resistant emerging as a potential modifier of resistance levels. These findings underscore the nuanced relationship between aminoglycoside exposure and soil microbial ecology, highlighting key factors like soil properties that may modulate microbial diversity outcomes.

### Implications for policy/management

The research suggests that exposure to aminoglycosides does not significantly alter the overall diversity (alpha diversity) of soil microbial communities. However, there may be relationships between soil pH, the aminoglycoside used, soil use, and microbial diversity. Additionally, exposure to these antibiotics appears to affect the proportion of bacteria that become resistant.

This evidence indicates that while aminoglycosides might not drastically change soil diversity in general, they could contribute to an increase in antibiotic-resistant bacteria. The uncertainty around these findings includes potential variations due to factors like the relationship between the exposure concentration and selection concentration. This information can inform decisions by highlighting the possible impact of aminoglycosides on microbial diversity depending on soil properties, but further research is needed to determine if this is a true modifier.

### Implications for research

pH, soil use, method of administration, and type of intervention emerged as potential modifiers of microbial diversity. Given the relationship between soil conditions and antibiotic adsorption, these findings highlight the need for further research exploring the connection between soil properties and microbial diversity. Additional research should be crafted towards understanding the role of antibiotic adsorption on the impact of aminoglycosides in soil.

The current evidence on the impact of aminoglycoside exposure on disease suppression in soil ecosystems is limited by several knowledge gaps. A major shortcoming is the scarcity of studies that directly investigate how aminoglycosides affect disease suppression. This gap highlights the need for targeted research focused on understanding the relationship between aminoglycoside exposure, microbial community dynamics, and disease outcomes in diverse soil types. Future studies should aim to address these gaps by exploring how these antibiotics influence disease suppression in agricultural contexts.

Another significant limitation is the exclusion of certain aminoglycosides—such as tobramycin, amikacin, and others—from the current literature, likely due to their limited use in agriculture. While these antibiotics are not commonly used, they may have distinct effects on soil microbial communities. Future research should broaden its scope to include these less commonly studied aminoglycosides to provide a more comprehensive understanding of their environmental impact.

Finally, many studies lack key data on confounding factors, such as soil composition, compost use, and nitrogen content, which are crucial for interpreting the effects of aminoglycosides. Inadequate control groups and incomplete data reporting further hinder the reliability of current findings. Future research should prioritize rigorous study designs with well-defined control groups and thorough reporting of experimental conditions. Addressing these gaps will strengthen the evidence base and improve our understanding of aminoglycoside effects on soil health and disease suppression.

## Electronic supplementary material

Below is the link to the electronic supplementary material.

Supplementary Material 1

Supplementary Material 2

Supplementary Material 3

Supplementary Material 4

Supplementary Material 5

Supplementary Material 6

## Data Availability

No datasets were generated or analysed during the current study.
